# An In-Hospital Mortality Risk Model for Elderly Patients Undergoing Cardiac Valvular Surgery Based on LASSO-Logistic Regression and Machine Learning

**DOI:** 10.3390/jcdd10020087

**Published:** 2023-02-17

**Authors:** Kun Zhu, Hongyuan Lin, Xichun Yang, Jiamiao Gong, Kang An, Zhe Zheng, Jianfeng Hou

**Affiliations:** 1 Cardiac Surgery Centre, Fuwai Hospital, Chinese Academy of Medical Sciences and Peking Union Medical College, Beijing 100037, China; 2 Department of Anesthesiology, Beijing Cancer Hospital, Peking University, Beijing 100142, China

**Keywords:** valvular heart disease, mortality risk, prediction models, LASSO-logistic regression, machine learning

## Abstract

Background: To preferably evaluate and predict the risk for in-hospital mortality in elderly patients receiving cardiac valvular surgery, we developed a new prediction model using least absolute shrinkage and selection operator (LASSO)-logistic regression and machine learning (ML) algorithms. Methods: Clinical data including baseline characteristics and peri-operative data of 7163 elderly patients undergoing cardiac valvular surgery from January 2016 to December 2018 were collected at 87 hospitals in the Chinese Cardiac Surgery Registry (CCSR). Patients were divided into training (N = 5774 [80%]) and testing samples (N = 1389 [20%]) according to their date of operation. LASSO-logistic regression models and ML models were used to analyze risk factors and develop the prediction model. We compared the discrimination and calibration of each model and EuroSCORE II. Results: A total of 7163 patients were included in this study, with a mean age of 69.8 (SD 4.5) years, and 45.0% were women. Overall, in-hospital mortality was 4.05%. The final model included seven risk factors: age, prior cardiac surgery, cardiopulmonary bypass duration time (CPB time), left ventricular ejection fraction (LVEF), creatinine clearance rate (CCr), combined coronary artery bypass grafting (CABG) and New York Heart Association (NYHA) class. LASSO-logistic regression, linear discriminant analysis (LDA), support vector classification (SVC) and logistic regression (LR) models had the best discrimination and calibration in both training and testing cohorts, which were superior to the EuroSCORE II. Conclusions: The mortality rate for elderly patients undergoing cardiac valvular surgery was relatively high. LASSO-logistic regression, LDA, SVC and LR can predict the risk for in-hospital mortality in elderly patients receiving cardiac valvular surgery well.

## 1. Introduction

Valvular heart diseases (VHDs) are some of the most common cardiovascular diseases in China. The number of patients requiring cardiac valvular surgery has been rising as the social structure of the population ages [1,2]. About 270,000 heart valve surgeries are performed each year worldwide, accounting for 20–35% of all cardiovascular surgeries [3]. The mortality rate during postoperative hospitalization is 2.6–6.8% in some high-income countries, such as the United States, and can be as high as 13.29% or more if multiple valvular surgery or combined CABG is performed at the same time [4]. In China, close to 80,000 heart valve surgeries are performed each year, while the postoperative mortality rate is nearly 2.3% [5,6]. The current increased incidence, surgical comorbidities, operative difficulties of VHDs and high risk of postoperative complications and mortality have brought a heavy medical burden on individuals, families and society.

With the increase in the incidence of VHDs and surgeries cases, the attention of surgeons and the mature application of research methods such as logistic regression, a series of risk prediction models were developed and applied to patients undergoing cardiac valvular surgery, such as the Society of Thoracic Surgeons National Cardiac Surgery Database score (STS-NCD score) [7], the European System for Cardiac Operative Risk Evaluation (EuroSCORE) [8,9] and the original Chinese CABG risk model (SinoSCORE) [10], based on the establishment of large databases and the analysis of risk factors for postoperative complications and mortality, but several problems still exist in poor discrimination, accuracy and applicability.

Compared with young and middle-aged patients, elderly patients have a much higher risk of mortality after cardiac valvular surgery [11]. With the increasing age of China’s population, the burden of VHDs in elderly patients has increased significantly.

Traditional statistical tools such as LASSO-logistic regression and machine learning are currently the most common methods for predictive model construction, and both have similar tasks that mainly include model parameter inference and data fitting or prediction. However, the focuses of these two studies are different. Statistics is more concerned with the confidence of inference or prediction, while machine learning is more concerned with the predictive effect of the model.

The different emphases also lead to many differences in the methodology of the research. The statistical society is concerned about the distribution of statistics, whether the hypothesis test is significant and whether the model fitting is reasonable. Machine learning is concerned with problems directly related to improving the prediction effect, such as how to design models or objective functions, how to train, how to improve the efficiency of algorithms, etc. We applied statistical methods and machine learning to better predict the risk of mortality in elderly patients undergoing cardiac valvular surgery.

To achieve continuous quality monitoring and improvement in adult cardiac surgery, the Chinese Cardiac Surgery Registry (CCSR) database was established in 2013 [12]. This study intends to identify risk factors after cardiac valvular surgery by analyzing the clinical data of elderly patients included in the CCSR database, construct prediction models using LASSO-logistic regression and machine learning algorithms to provide new ideas for mortality risk assessment.

## 2. Materials and Methods

### 2.1. Study Population

The CCSR database includes data from consecutive patients undergoing cardiac surgery at 87 participating centers located in nearly all provinces and directly controlled municipalities in China [12]. Preoperative risk factors and in-hospital deaths are recorded for each patient.

**Figure 1:**
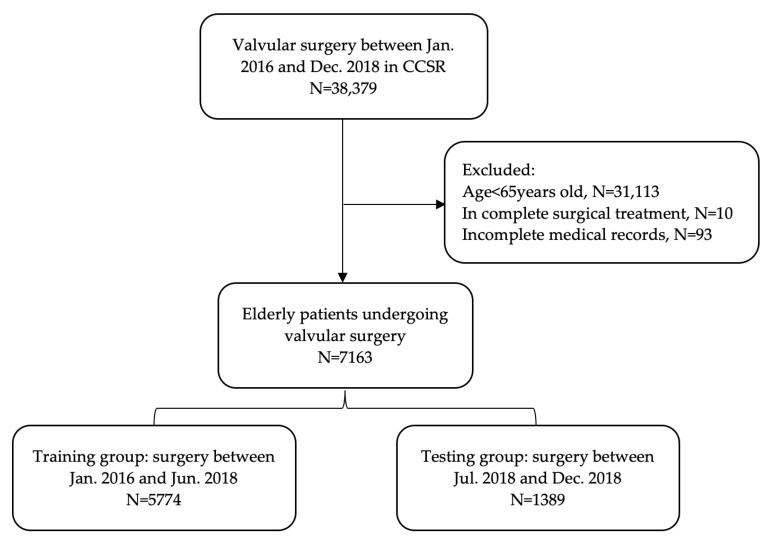
Flow chart of patient enrollment and final sample division.

From January 2016 to December 2018, 7163 elderly patients undergoing cardiac valvular surgery included in the CCSR database were selected for the study, including 3939 males and 3224 females with a mean age of 69.8 (SD 4.5) years (Figure 1). Inclusion criteria were patients who underwent cardiac valvular surgery, including mitral, tricuspid, aortic and pulmonary valve surgery, combined or uncombined with other cardiovascular surgery; greater than or equal to 65 years of age. Exclusion criteria: incomplete surgical treatment; incomplete medical records.

### 2.2. Definitions of Parameters

In-hospital mortality in our research was defined as all-cause death occurring between the surgery and hospital discharge.

Definitions of demographics and clinical variables are shown in Table 1.

### 2.3. Data Collecting

Recorded variables included clinical, perioperative and laboratory data. All patients were examined using preoperative echocardiography to record left ventricular ejection fraction (LVEF), left ventricular end diastolic diameter (LVEDD), left atrial dimension (LAD) and valvular lesions including aortic stenosis (AS), aortic insufficiency (AI), mitral stenosis (MS), mitral insufficiency (MI), tricuspid stenosis (TS), tricuspid insufficiency (TI), pulmonary stenosis (PS) and pulmonary insufficiency (PI). Candidate risk factors for the model recorded as clinical data included: age, gender, body mass index (BMI), comorbidities, preoperative medications, tobacco, alcohol, nutrition, NYHA classification and Canadian Cardiovascular Society (CCS) angina classification. Perioperative data studied included surgical status, surgical approach, CPB time, aortic cross-clamp (ACC) time, mechanical ventilation time, estimated blood loss, blood transfusion including red blood cell (RBC) and fresh frozen plasma (FFP), hospitalization time, ICU stays and complications. Laboratory results included total cholesterol (TC), low density lipoprotein (LDL), fasted blood glucose (FBG) and serum creatinine (SCr). CCr was calculated using the Cockcroft-Gault formula. Comorbidities included hypertension, diabetes mellitus, dyslipidemia, cerebrovascular accident, chronic kidney disease (CKD), chronic obstructive pulmonary disease (COPD), peripheral vascular disease (PVD), cardiac arrhythmias, coronary artery disease (CAD), prior myocardial infarction (MI), prior percutaneous coronary intervention (PCI), prior heart failure (HF) and prior cardiac surgery. The primary study endpoint was in-hospital mortality, defined as all-cause in-hospital death after cardiac valvular surgery.

### 2.4. Statistical Analysis

Continuous variables were presented as mean ± standard deviation (SD) if they obeyed a normal distribution or median (quartiles) besides and were compared using Student’s *t*-test and Mann–Whitney test. Categorical variables were presented as frequencies (percentages%) and compared using Chi-square and Fisher’s exact tests. The Kolmogorov–Smirnov test was adopted for normality testing. All *p* values were two-tail and *p* < 0.05 was considered significant. Data were analyzed using SPSS version 26.0 (IBM Corp., Armonk, NY, USA) and GraphPad Prism version 9.3.1 (GraphPad Software, San Diego, CA, USA). R version 4.2.1 “rms”,“CBCgrps”,“caret”,“glmnet” package (R Foundation for Statistical Computing, Vienna, Austria) was used to build the LASSO-logistic prediction model and Python version 3.10 (Python Software Foundation, Wilmington DE, USA) was used to build the machine learning models.

At the traditional statistical analysis level, we replaced the columns with continuous values in the dataset with the average value of the remaining values in the column. If the missing value came from the classification column (string or value), we replaced the missing value with the most common category. For data variables with longitudinal behavior (time variables in this study), the last valid observation value was used to fill in the missing value.

At the level of machine learning, this study first discarded samples with missing values greater than 20%, which means that when a patient’s clinical data have 20% or more missing, we excluded them from the data set. Secondly, we used the deep learning algorithm to predict the remaining missing values and the filling method of Datawig database (https://github.com/awslabs/datawig) (accessed on 21 April 2021).

In training set, LASSO regression was used to screen variables. We utilized ten-fold cross-validation to select the penalty term, λ. The binomial deviance was computed for the test data as measures of the predictive performance of the fitted models. Logistic regression analysis was used with the “Forward LR” method. Subsequently, we constructed a nomogram based on the logistic regression model using the “rms” package. According to the results of the regression, multiple line segments were drawn according to a specific proportion, and the incidence risk or survival probability of an individual was easily calculated by making the graph.

In addition, ML prediction models were established using 11 algorithms: Adaboost, BernoulliNB, decision tree (DT), gradient boosting (GB), K-nearest neighbor (KNN), linear discriminant analysis (LDA), support vector classification (SVC) and logistic regression (LR), random forest (RF), stochastic gradient descent (SGD) and extreme gradient boosting (XGBoost). Before this, the dimension of extracted features was reduced using the Select K Best (K-Best) algorithm and traditional least absolute shrinkage and the selection operator (LASSO) algorithm. Finally, the relevant parameters of these selected features were used to build a machine learning model. We used area under the curve (AUC) to evaluate the discriminatory performance of the model, the Hosmer–Lemeshow goodness-of-fit test, calibration curve and brier score to assess model calibration. In addition, EuroSCORE II model was used to compare prediction performance with our model through Delong test.

## 3. Results

### 3.1. Perioperative Data

A total of 7163 elderly patients undergoing cardiac valvular surgery between January 2016 and December 2018 met the study criteria and were included in this analysis (Table 2). There were 3939 (55.0%) males and 3224 (45.0%) females, with a mean age of 69.8 (SD 4.5) years. The patients had a mean preoperative LVEF of 59.6 (SD 8.8)%, LVEDD of 54.2 (SD 10.5) mm and LAD of 47 (SD11.0) mm. In total, 3757 (52.5%) of these patients underwent aortic valve surgery, 4354 (60.8%) underwent mitral valve surgery, 2623 (36.6%) underwent tricuspid valve surgery and one patient underwent pulmonary valve surgery, of which 1268 (17.7%) underwent a combined valve procedure. Grouping by years, there were 2073 cases (28.9%) in 2016, 2325 cases (32.5%) in 2017 and 2765 cases (38.6%) in 2018.

In-hospital mortality in the aggregate cohort was 4.05% (290/7163). With regard to preoperative factors, patients dead and non-dead differed statistically significantly according to age, tobacco, dyslipidemia, cerebrovascular accident, prior heart failure, NYHA and CCS classification, cardiac arrhythmias, prior myocardial infarction, prior cardiac surgery, SCr, CCr, TC, LDL, FBG, LVEF, MS, MI grades and TI grades. As for intraoperative characters, mortality was significantly associated with CPB time, ACC time, combined CABG and blood transfusion (*p* < 0.05).

### 3.2. Screening Results of Variables of the Prediction Models

According to the result of single-factor analysis shown (*p* < 0.1 was considered partly significant and these variables were chosen) and possible clinical significance, screening factors for prediction models were as follows: age, gender, BMI, tobacco use, hypertension, diabetes mellitus, dyslipidemia, COPD, PVD, prior cerebrovascular accident, prior HF, CCS class, NYHA class, atrial flutter/atrial fibrillation, prior MI, prior cardiac surgery, SCr, CCr, TC, LDL, FBG, LVEF, LVEDD, CPB time, ACC time, combined CABG, etc.

### 3.3. Establishment of the LASSO-Logistic Regression Prediction Model

**Figure 2:**
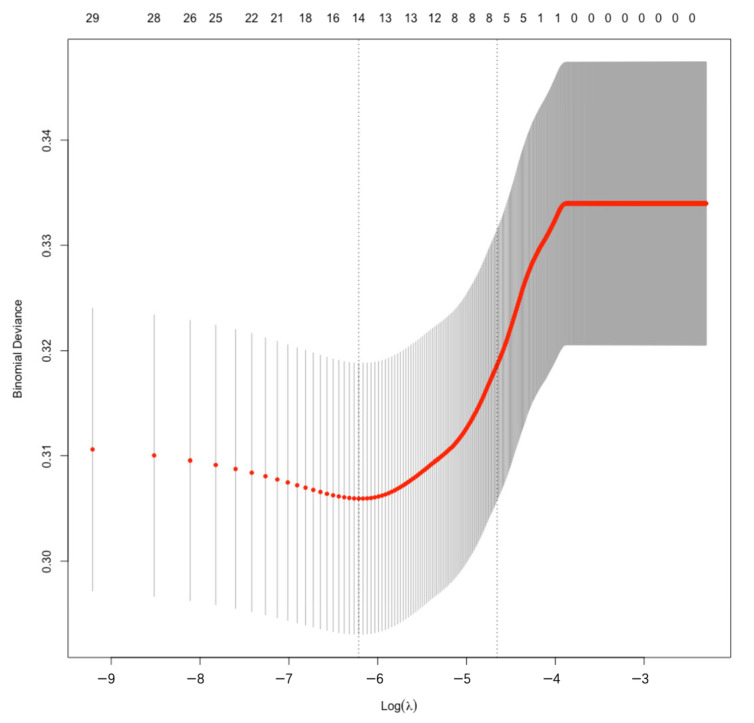
Log(λ) and the error model. The 2 dashed lines in Figure 2 indicate lambda.min and lambda.1se, respectively. Lambda.min denoted the value of λ when the model error was minimal. Lambda.1se denoted the model error within a standard error range of λ.

**Figure 3:**
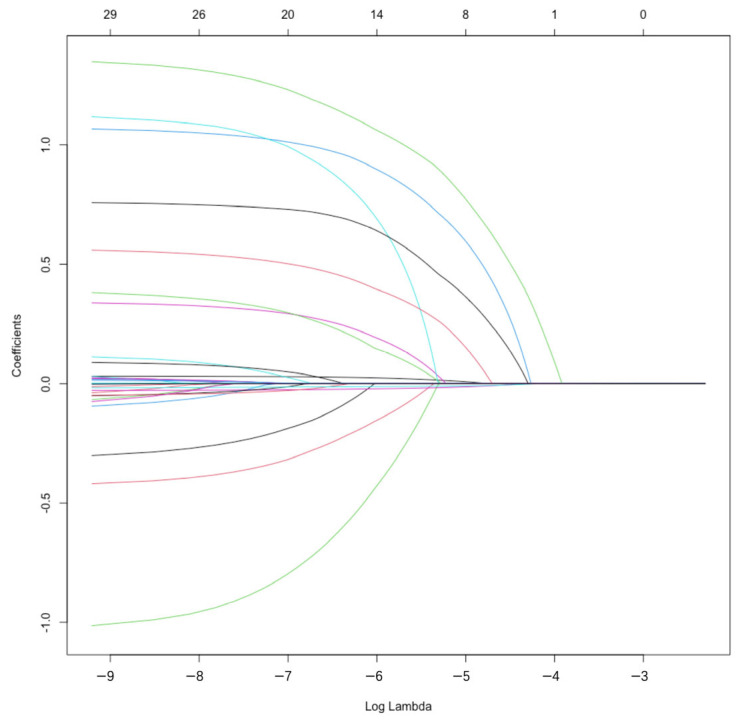
Log(λ) and the LASSO regression coefficient.

In the training sample, in-hospital mortality was used as the dependent variable, the variables were screened using the LASSO regression algorithm, and the best λ value was selected by 10 folds cross-validation (Figure 2 and Figure 3). The 2 dashed lines in Figure 2 indicate lambda.min and lambda.1se, respectively. Lambda.min denoted the value of λ when the model error was minimal. Lambda.1se denoted the model error within a standard error range of λ. At this point, the fit was guaranteed while incorporating the least number of variables to obtain the most streamlined prediction model. LASSO regression screening variables were age, LVEF, combined CABG, CCr, prior cardiac surgery, CPB time and NYHA class (Table 3).

The LASSO-logistic model equation is Logit *p* = −5.669 + 0.036 × age(years) + 0.928 × prior cardiac surgery − 0.026 × LVEF(%) + 0.01 × CPB time(min) + 0.389 × combined CABG + 0.328 × NYHA class − 0.021 × CCr(mL/min/1.73 m^2^).

**Figure 4:**
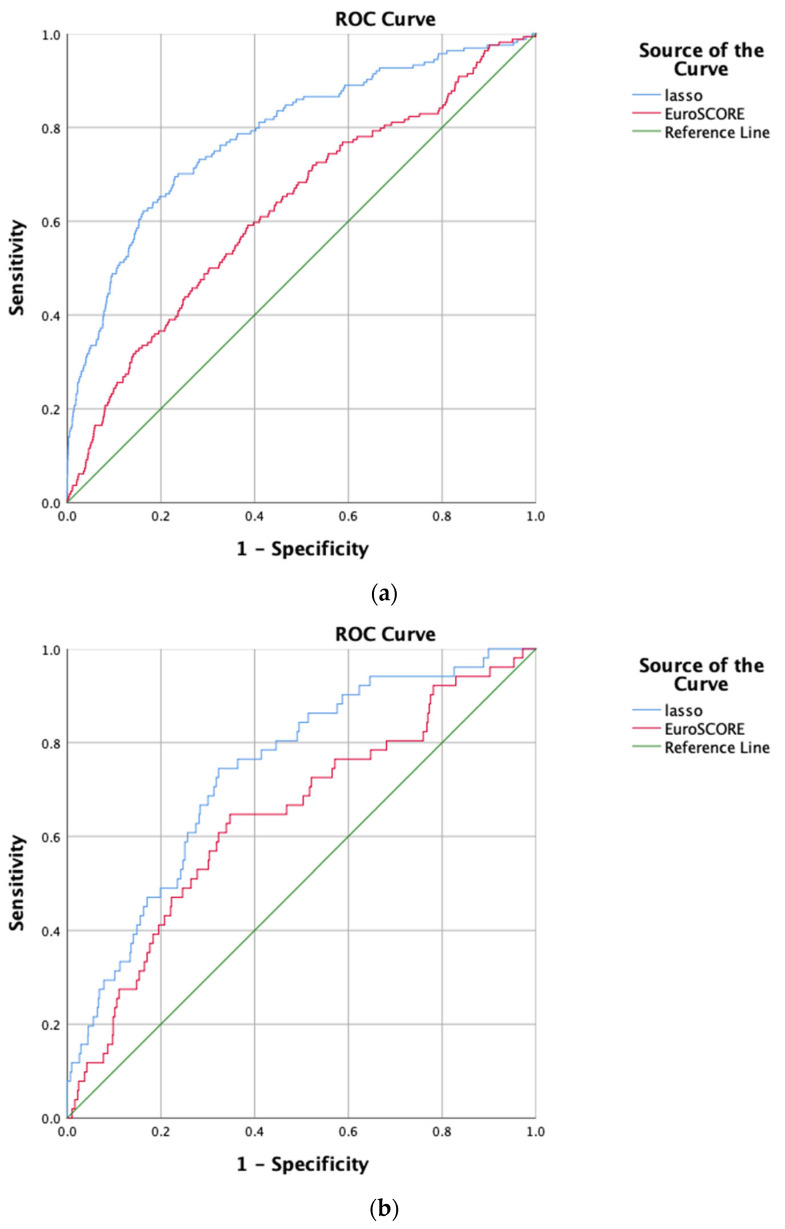
(**a**) ROC of LASSO-logistic regression and the EuroSCORE II in training cohort. (**b**) ROC of LASSO-logistic regression and the EuroSCORE II in testing cohort.

**Figure 5:**
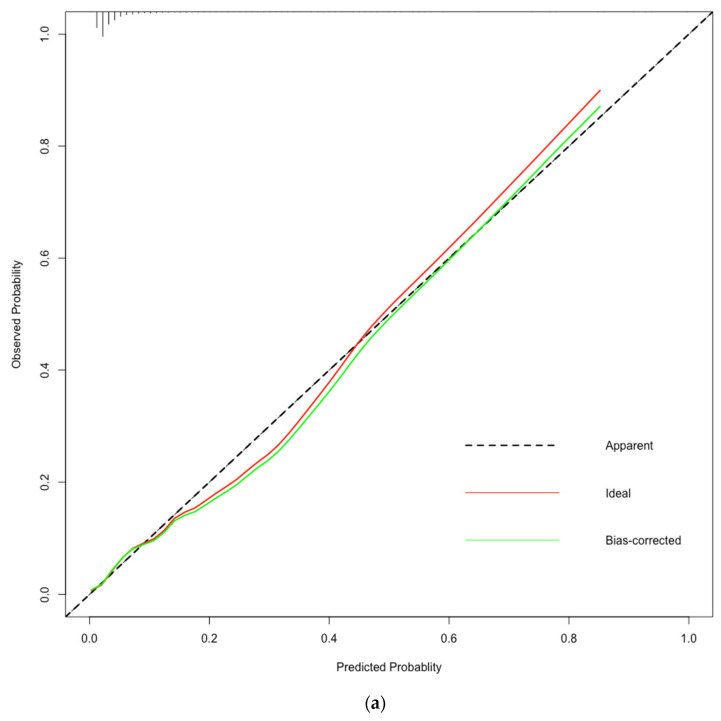

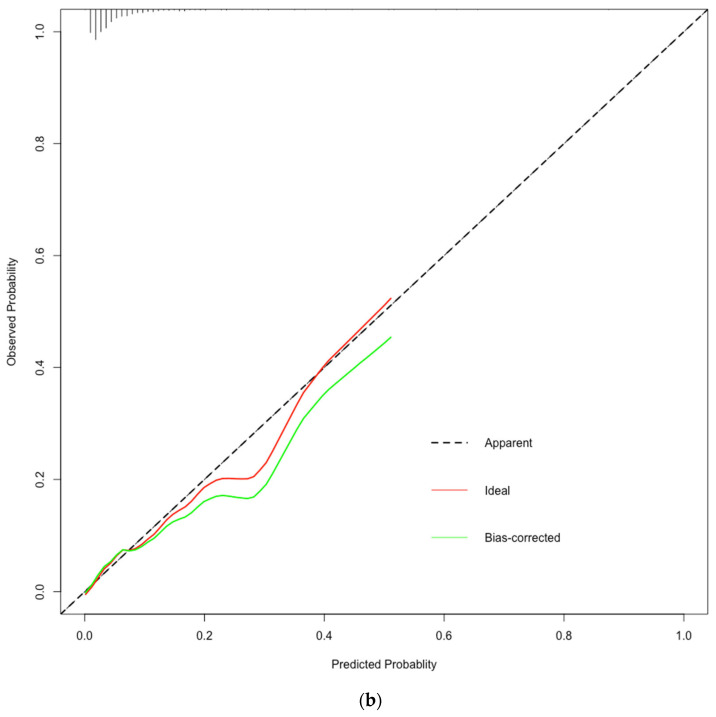
(**a**) Calibration curve of LASSO-logistic regression and the EuroSCORE II in training cohort. (**b**) Calibration curve of LASSO-logistic regression and the EuroSCORE II in testing cohort.

In the training data, the AUC of the LASSO-logistic regression prediction model was 0.785 (95% CI:0.746–0.824) (Figure 4a). The Hosmer–Lemeshow χ^2^ was 10.731, *p* value was 0.217 and the calibration curve of the prediction model was close to the standard curve (Figure 5a), suggesting that the model predicted the risk of in-hospital mortality after heart valve surgery with high accuracy and good agreement with the actual risk of occurrence. The AUC of the EuroSCORE II prediction model was 0.627 (95%CI: 0.582–0.672).

In the testing sample, the AUC of this model was 0.739 (95% CI:0.673–0.805) (Figure 4b). The Hosmer–Lemeshow χ^2^ was 6.64, *p* value was 0.576 and the calibration curve of the prediction model was close to the standard curve (Figure 5b). The AUC of the EuroSCORE II prediction model in testing sample was 0.642 (95%CI: 0.562–0.722). The LASSO-logistic regression model outperformed the EuroSCORE II prediction model in terms of discrimination and calibration.

**Figure 6:**
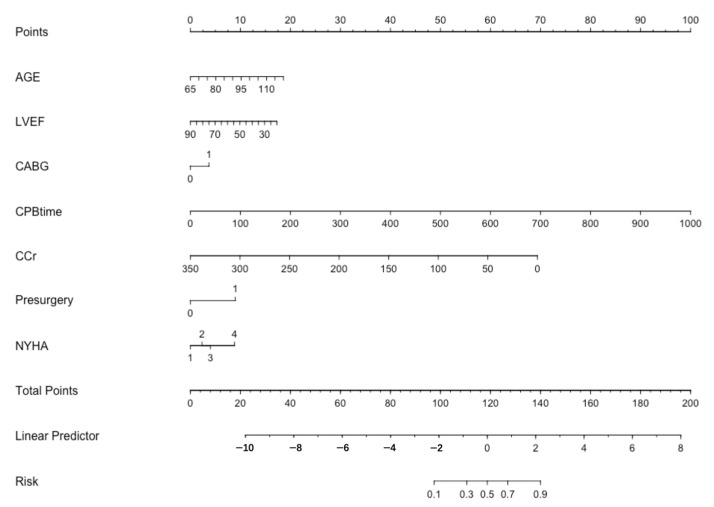
Nomogram prediction model of in-hospital mortality for elderly patients undergoing cardiac valvular surgery. Each variable corresponds to a risk score, and the total score is obtained by summing all scores to the corresponding risk of mortality.

**Figure 7:**
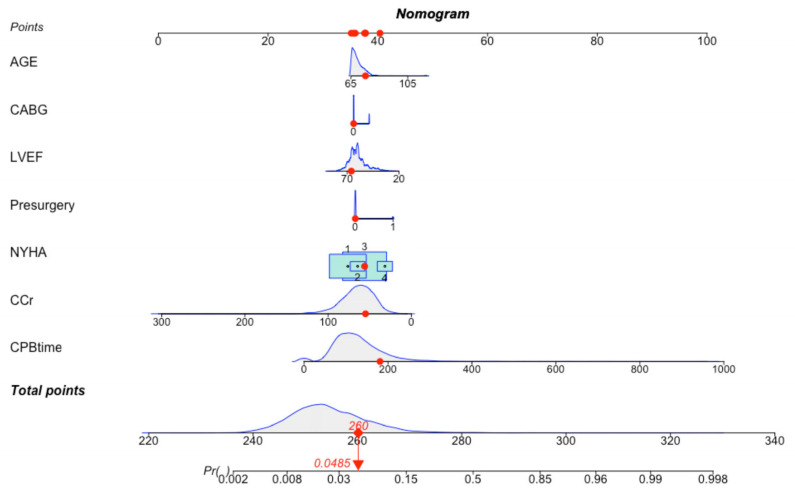
One example of nomogram. Enter the values of 7 key variables to predict the risk of in-hospital mortality and show the contribution of each value to the outcome.

Variables from the LASSO-logistic regression model were utilized to build a nomogram model (Figure 6). In the case of this patient, aged 75 years, with no prior cardiac surgery, a preoperative LVEF of 66.0%, a CPB time of 181 min, non-combined CABG, a preoperative CCr of 55.1 mL/min/1.73 m^2^ and NYHA class III, a risk score of 260 points and a risk of mortality of 4.85% could be calculated according to the nomogram model (Figure 7).

### 3.4. Establishment of ML Prediction Models

**Figure 8:**
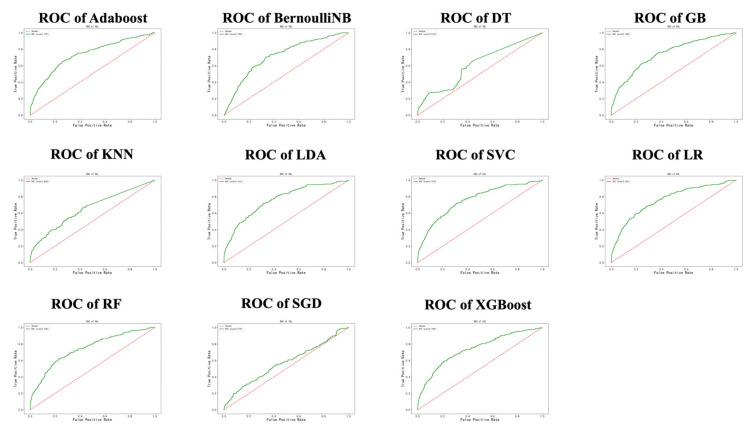
ROCs of ML prediction models in training cohort.

**Figure 9:**
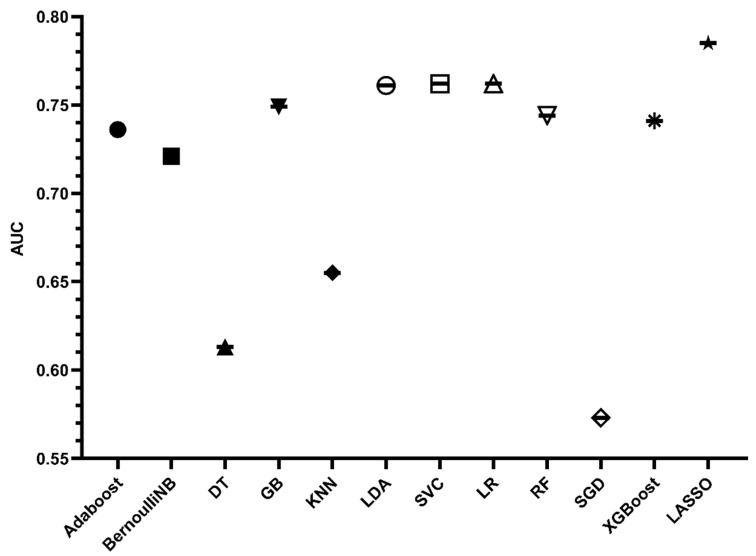
AUCs of nomogram and ML prediction models in training cohort.

**Figure 10:**
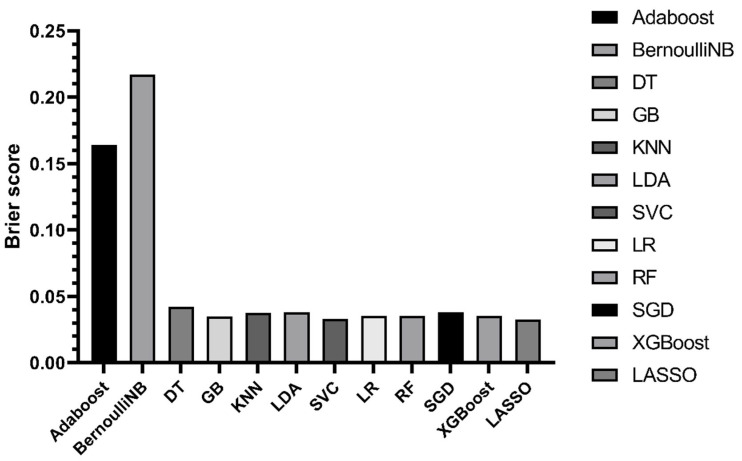
Brier scores of nomogram and ML prediction models in training cohort.

In the training data, model establishments and internal validation were performed using the 10 folds cross validation method. The ROC is shown in Figure 8 and Figure 9 and the Brier score is shown in Figure 10. The results showed that LDA, SVC and LR had the largest AUC and the lower Brier score, which suggests superior discrimination and calibration of other ML prediction models. In addition, we observed a larger AUC of the nomogram than other ML models and a similar Brier score to the LDA, SVC and LR models.

**Figure 11:**
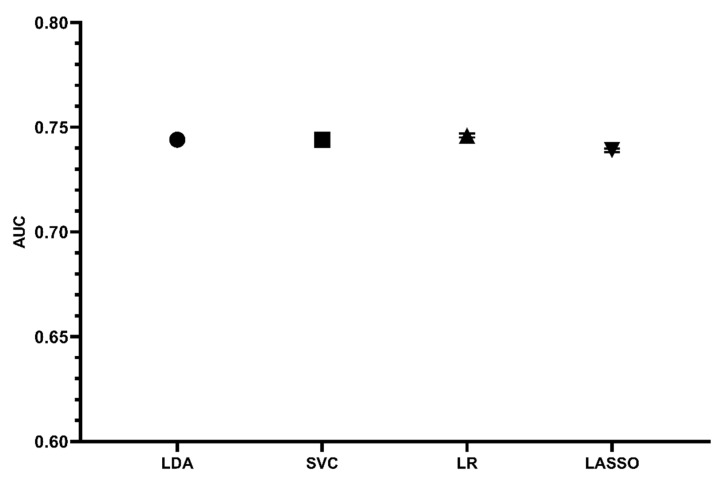
AUCs of nomogram and ML prediction models in testing cohort.

**Figure 12:**
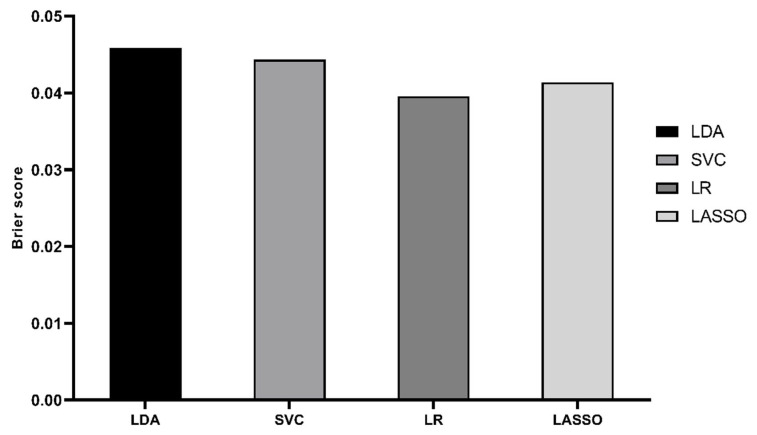
Brier score of nomogram and ML prediction models in testing cohort.

In the testing cohort, we externally validated the nomogram model and the machine learning algorithms such as LDA, SVC and LR with the best prediction performances in the training cohort (Figure 11 and Figure 12). Four prediction models were found to have satisfying discrimination and calibration, demonstrating their promising application in assessing the mortality risk of elderly patients after cardiac valvular surgery.

## 4. Discussion

VHDs are increasingly becoming an important public health problem and have a higher overall prevalence and percentage of patients requiring surgical treatment [1,2]. Degenerative and functional lesions are the main causes in high-income countries, while rheumatic lesions are still the main cause in low- and middle-income countries [1]. Shengshou Hu et. al. released an update of the Annual Report on Cardiovascular Health and Disease in China 2021, showing that the number of cardiovascular diseases in China is about 330 million, of which the prevalence of VHDs is 3.8%, with about 25 million people affected [6]. The establishment of a cardiac valvular surgery risk assessment model is of great significance for the accurate identification of high-risk cases, adequate assessment of surgical risks, targeted perioperative management and comprehensively improving the diagnosis and treatment level.

The prevalence of VHDs increases significantly with age, with a prevalence of about 0.7–2.1% in people aged 18–54 years and can be as high as 7.6–15.9% in elderly patients aged 65 years or elder [13]. With the increasing age of China’s population, the burden of VHDs and the number of patients requiring surgical treatment, especially elderly patients, has increased significantly. Compared with young and middle-aged patients, elderly patients have a much higher prevalence of comorbidities such as hypertension, diabetes mellitus, cerebrovascular disease, chronic kidney disease and atrial fibrillation, a weaker ability to tolerate cardiopulmonary bypass surgery, more complex pathologic anatomy such as valve annulus calcification, leaflet thickening and leaflet prolapse, a higher risk of postoperative death and related complications [13,14]. The perioperative management of elderly patients undergoing cardiac valvular surgery requires more clinical attention, and there is a lack of corresponding mortality risk prediction models and studies. Therefore, this study aims to establish a risk prediction model for postoperative mortality in elderly patients aged 65 years or older and to provide more accurate treatment plans for them.

The high postoperative mortality rate in elderly patients undergoing cardiac valvular surgery has been of great concern to cardiothoracic surgeons. The in-hospital mortality rate in the aggregate cohort was approximately 4.05% in our research, which was significantly higher than the overall population mortality rate (2.16–2.64%) and slightly better than the 8–20% mortality rate reported in previous studies [13,15]. Yoshida et al. found that the in-hospital mortality rate was 9.6% in patients 65 years or older, whereas in patients younger than 65 years, the rate was 3.2% [15]. The analysis by Susheel K. Kodali et al. found that in-hospital mortality in patients over 80 years undergoing cardiac valvular surgery could be as high as 20%, which may be higher if multiple valves were involved or combined with CABG surgery [13]. In our study, the mean age of the patients included was 69.8 years and 4470 patients (62.4%) were in the 65–70 range. Thus, the low mortality rate may be due to the improvement of large cardiac centers. On the one hand, Zhan Hu et al. found that advances in cardiac surgical care and the Chinese government’s efforts to improve population health have helped to decrease surgical mortality over time and existing risk models may now overestimate surgical mortality and poorly discriminate between patients [16]. On the other hand, patients included in our study were not too old and had a relatively low risk of mortality. In the study from Zhuge, 45.29% of patients over 60 years with mitral valve insufficiency were not treated surgically, compared with 10% in patients over 80 years. Older age, impaired LVEF, lower regurgitation grade, EuroSCORE II high risk stratification and having diabetes were factors most significantly associated with surgery denial among elderly Chinese inpatients with MR [17]. For elderly patients, surgical decisions are made more cautiously.

Risk factors for mortality after cardiac valvular surgery have been a focus and hot topic in cardiovascular surgery research. Previous studies have shown that plenty of risk factors seemed to be strong factors associated with mortality such as age, LVEF, BMI, renal function, combined CABG, NYHA class, etc. [7,9,18]. Seven variables such as age, LVEF, CCr, etc., were included in our nomogram model, which maximized the simplicity and efficiency. Compared with previous studies, our model included two relatively new variables with less incorporation of previous predictive models. 

First, our study showed that prior cardiac surgery was one of the independent risk factors (OR = 2.529, 95% CI = 1.572–4.070, *p* < 0.001). For patients overall, the proportion of patients with prior cardiac surgery was 5.5% and it was 12.1% of patients in the death group. Most patients had undergone valvular surgery, with a small percentage having undergone CABG. In 2004, Nowicki et al. analyzed data from the NNECDSG database of 8943 cardiac valvular surgery patients from 1991 to 2001, and applied logistic regression analysis to develop a prediction model for the risk of in-hospital mortality for aortic and mitral valve surgery, incorporating prior cardiac surgery as one risk factor [19]. Because of the severe pleuropericardial adhesions resulting from the initial procedure, reoperation is related to greater difficulty, more blood loss, longer operative time and greater risk of cardiac injury, with the resulting increased risk of postoperative death being easily understood. With improved overall life expectancy and survival after cardiac surgery, the persistence of coronary artery disease, the extensive use of bioprosthetic heart valves and the rapid evolution of mitral valvuloplasty, the number of patients undergoing cardiac reoperation increases continuously. Lin H. et al. found that the proportion of mitral valvuloplasty in the CCSR database showed a rapid increase, rising 11.9% over three years [20]. It reminds us of the need to pay more attention to patients undergoing reoperative cardiac surgery, with adequate preoperative evaluation and preparation, intraoperative use of applicable accesses, intubation and more appropriate surgical equipment to reduce the risk of complications and mortality [1].

Second, the prediction model incorporated the lesser studied CPB time, which is an important controllable factor in valvular surgery. The mean CPB time was 196.4 min in the death group, while it was 126.4 min in the non-death group; every minute counts when the aortic was clamped during surgery (OR = 1.010, 95% CI: 1.009–1.012, *p* < 0.001). Stefano Salis et al. analyzed the data of 5006 patients undergoing CPB surgery and found that the CPB time was an independent risk factor for postoperative death (OR = 1.57, 95% CI:1.43–1.73, *p* < 0.0001) [21]. In addition, the increased CPB time leads to a significant increase in the incidence of complications such as renal, respiratory and neurological complications and multi-organ dysfunction and multiple blood transfusions. CPB time is currently considered to be associated with the activation of the inflammatory cascade response caused by the release of various inflammatory mediators, leading to an increased risk of organ dysfunction and mortality [22,23,24]. On the other hand, increased CPB time implies prolonged operative time and assist circulation time, which can partly reflect the difficulty of surgery and the more critical condition of the patient.

In this study, we used LASSO-logistic regression and machine learning analysis for modeling. The constructed prediction model outperformed the traditional EuroSCORE II in the training and testing cohort in terms of discrimination and calibration (*p* < 0.05) and was more suitable for the assessment of mortality risk in elderly patients after cardiac valvular surgery. This study has the following advantages over the EuroSCORE II: 1. previous prediction models such as STS-NCD score and EuroSCORE are based on data from Western populations [7,9], and there may be significant differences in disease features, therapeutic strategies and surgical techniques in different regions [7]. Our study was supported by the CCSR database. Established in 2013, CCSR is a nationwide multicenter registry that provides a platform for risk assessment, outcome evaluation and quality improvement for adult cardiac operations in mainland China with the advantages of a border range of participating centers, strong representation and high-quality data [12,16]. Our study reflected the cardiac surgical levels in China better and was more accurate in predicting the risk of mortality after valve surgery. 2. Based on clinical data from patients more than 10 years ago, Nashef et al. proposed the EuroSCORE I scoring system in 1999 [8], which was updated to EuroSCORE II in 2012 [9]. As cardiac surgery technology has improved and disease characteristics have changed over the past decade, the risks and predictive variables of mortality from valve surgery in previous studies are no longer representative of current clinical practice. 3. A total of 18 predictive variables are included in EuroSCORE II [9], while seven variables are included in our prediction model, which is easier to use. Overall, our predictive model may serve as a better assessment tool for evaluating the risk of mortality after cardiac valvular surgery in the Chinese elderly population, and its potential applicability could be investigated in other regions or populations in the future.

Machine learning has been a cutting-edge interdisciplinary study direction in recent years and is increasingly used in clinical settings [25,26]. Machine learning has unique advantages in handling large sample data, complex data and personalized assessments [27]. ML approaches have been applied in medical fields as an emerging technological means, such as imaging diagnosis, pharmaceutical research and the establishment of prediction models. A series of studies have already applied machine learning algorithms to the field of risk prediction for cardiac surgery, including mortality and postoperative complications such as acute kidney injury, myocardial infarction and readmission [28,29,30,31]. Allyn et al. found that machine learning algorithms were far more accurate than EuroSCORE II and logistic regression in predicting in-hospital mortality after elective cardiac surgery [32]. Machine learning involves a series of algorithms, and different algorithms have different learning ways and application scenarios, so we need to evaluate the prediction performance of each machine algorithm and select the most suitable prediction model. Our study found that the application of LDA, SV and LR algorithms for postoperative mortality risk assessment demonstrated excellent predictive performance by comparing AUC values and Brier scores. We introduced machine learning algorithms for a preliminary analysis to explore their great potential for risk assessment. The advantages of machine learning algorithms may become more apparent in the future as the research continues and the sample size, as well as the collection of variables, expands.

### Limitations

Our study has certain limitations, including the following aspects: first, our primary outcome was in-hospital mortality after valvular surgery due to the limitations of the CCSR database, rather than 30-day mortality, which was widely used. Second, the LASSO-logistic regression model incorporates an intraoperative variable of CPB time and is therefore limited in clinical application. Third, our study is limited by the lack of follow-up data on survival and other primary outcomes, and we await more research and spread in the future.

## 5. Conclusions

The mortality rate for elderly patients undergoing cardiac valvular surgery was relatively high. LASSO-logistic regression, LDA, SVC and LR can predict the risk for in-hospital mortality in elderly patients receiving cardiac valvular surgery well.

## Figures and Tables

**Table 1:** Definitions of risk factors for univariate analysis.

Variable	Definition
Age	-
Gender	-
BMI	Body mass index
Tobacco use	-
Hypertension	Documented past history or SBP > 140 mmHg and/or DBP > 90 mmHg
Diabetes mellitus	Documented past history or fulfilled the criteria of WHO 1999
Dyslipidemia	Documented past history, total cholesterol > 5.72 mmol/L or triglyceride > 1.70 mmol/L
COPD	Long-term use of bronchodilators or steroids for lung disease
Prior vascular surgery	Documented past history
Prior cerebrovascular accident	Documented past history
Prior HF	Documented past history
CCS class	-
NYHA class	-
Atrial flutter/Atrial fibrillation	Documented past history
Prior MI	Documented past history
Prior vascular surgery	1 or more previous major cardiac operations involving opening the pericardium
SCr	-
CCr	Calculated using the Cockcroft-Gault formula
Total cholesterol	-
LDL	-
FBG	-
LVEF	-
LVEDD	-
CPB time	-
ACC time	-
Combined CABG	Combined with CABG surgery
In-hospital postoperative mortality	All-cause mortality

BMI, body mass index; SBP, systolic blood pressure; DBP, diastolic blood pressure; WHO, World Health Organization; COPD, chronic obstructive pulmonary disease; HF, heart failure; CCS, Canadian Cardiovascular Society; NYHA, New York Heart Association; MI, myocardial infarction; SCr, serum creatinine; CCr, creatinine clearance rate; LDL, low density lipoprotein; FBG, fasted blood glucose; LVEF, left ventricular ejection fraction; LVEDD, left ventricular end-diastolic diameter; CPB, cardiopulmonary bypass; ACC, aortic cross-clamp; CABG, coronary artery bypass grafting.

**Table 2:** Patient demographics and clinical features.

Variables	Overall (N = 7163)	Death (N = 290)	Non-Death (N = 6873)	*p* Value *
Baseline characteristics				
Age (years)	69.8 ± 4.5	71.0 ± 5.2	69.7 ± 4.5	<0.001
65–70	4470	142 (49.0%)	4328 (63.0%)	0.368
70–75	1931	88 (30.3%)	1843 (26.8%)	0.210
≥75	762	60 (20.7%)	702 (10.2%)	0.422
Male	3939 (55.0%)	158 (54.5%)	3781 (55.0%)	0.859
BMI (kg/m^2^)	23.23 (21.09,25.35)	22.86 (20.75,25.05)	23.24 (21.10,25.38)	0.133
Tobacco use	2994 (41.8%)	97 (33.4%)	2897 (42.2%)	0.003
Preoperative factors				
Hypertension	3165 (44.2%)	142 (49.0%)	3023 (44.0%)	0.094
Diabetes mellitus	850 (11.9%)	44 (15.2%)	806 (11.7%)	0.076
Dyslipidemia	1435 (20.0%)	38 (13.1%)	1398 (20.3%)	0.003
CKD	258 (3.6%)	13 (4.5%)	245 (3.6%)	0.411
COPD	150 (2.1%)	8 (2.8%)	142 (2.1%)	0.420
PVD	226 (3.2%)	14 (4.8%)	212 (3.1%)	0.096
Prior cerebrovascular accident	455 (6.4%)	34 (11.7%)	421 (6.1%)	<0.001
Prior HF	550 (7.7%)	41 (14.1%)	509 (7.4%)	<0.001
CCS class				0.002
None	3960 (55.3%)	152 (52.4%)	3808 (55,4%)	
CCS I	1027 (14.3%)	35 (12.1%)	992 (14.4%)	
CCS II	1067 (14.9%)	35 (12.1%)	1032 (15.0%)	
CCS III	608 (8.5%)	35 (12.1%)	573 (8.3%)	
CCS IV	70 (1.0%)	7 (2.4%)	63 (0.9%)	
NYHA class				<0.001
NYHA I	415 (5.8%)	18 (6.2%)	397 (5.8%)	
NYHA II	2662 (37.2%)	72 (24.8%)	2590 (37.7%)	
NYHA III	3496 (48.8%)	154 (53.1%)	3342 (48.6%)	
NYHA IV	452 (6.3%)	42 (14.5%)	410 (6.0%)	
Cardiac arrhythmias	1866 (26.1%)	91 (31.4%)	1775 (25.8%)	0.035
Ventricular tachycardia	33 (0.5%)	4 (1.4%)	29 (0.4%)	0.005
Ventricular fibrillation	15 (0.2%)	0	15 (0.2%)	0.469
Atrial flutter/atrial fibrillation	1745 (24.4%)	86 (29.7%)	1659 (24.1%)	<0.001
Atrioventricular block	51 (0.7%)	2 (0.7%)	49 (0.7%)	0.824
Prior MI	240 (3.4%)	18 (6.2%)	222 (3.2%)	0.006
Prior PCI	214 (3.0%)	8 (2.8%)	206 (3.0%)	0.815
Prior cardiac surgery	395 (5.5%)	35 (12.1%)	360 (5.2%)	<0.001
Prior CABG	35 (0.5%)	3 (1.0%)	32 (0.5%)	0.039
Prior valvular surgery	255 (3.6%)	21 (7.2%)	234 (3.4%)	<0.001
Prior congenital heart disease surgery	7 (0.1%)	1 (0.3%)	6 (0.1%)	0.058
Prior vascular surgery	10 (0.1%)	2 (0.7%)	8 (0.1%)	0.083
Others	65 (0.9%)	5 (1.7%)	60 (0.9%)	0.017
SCr (umol/L)	84.6 ± 34.2	99.7 ± 49.6	84.0 ± 33.2	<0.001
CCr (ml/min/1.73 m^2^)	63.6 ± 19.4	54.8 ± 19.4	64.0 ± 19.3	<0.001
TC (mmol/L)	4.12 (3.45,4.83)	3.88 (3.28,4.63)	4.12 (3.47,4.84)	0.001
LDL (mmol/L)	2.47 (1.94,3.05)	2.34 (1.86,2.87)	2.47 (1.94,3.06)	0.021
FBG (mmol/L)	5.6 ± 1.6	5.9 ± 2.1	5.5 ± 1.6	0.001
LVEF (%)	59.6 ± 8.8	56.2 ± 11.0	59.7 ± 8.6	<0.001
LVEDD (mm)	54.2 ± 10.5	53.5 ± 12.4	54.2 ± 10.4	0.388
LAD (mm)	47.0 ± 11.0	48.5 ± 11.6	46.9 ± 10.9	0.062
AS	1912 (26.7%)	66 (22.8%)	1846 (26.9%)	0.122
AI grades				0.357
None	3195 (44.6%)	118 (40.7%)	3077 (44.8%)	
Mild	1649 (23.0%)	77 (26.6%)	1572 (22.9%)	
Moderate	1402 (19.6%)	54 (18.6%)	1348 (19.6%)	
Severe	917 (12.8%)	41 (14.1%)	876 (12.7%)	
MS	1485 (20.7%)	79 (27.2%)	1406 (20.5%)	0.005
MI grades				0.015
None	2575 (35.9%)	83 (28.6%)	2492 (36.3%)	
Mild	1407 (19.6%)	52 (17.9%)	1355 (19.7%)	
Moderate	1653 (23.1%)	81 (27.9%)	1572 (22.9%)	
Severe	1528 (21.3%)	74 (25.5%)	1454 (21.2%)	
TS	18 (0.3%)	2 (0.7%)	16 (0.2%)	0.128
TI grades				0.004
None	3268 (45.6%)	110 (37.9%)	3158 (45.9%)	
Mild	1950 (27.2%)	79 (27.2%)	1871 (27.2%))	
Moderate	1367 (19.1%)	64 (22.1%)	1303 (19.0%)	
Severe	578 (8.1%)	37 (12.8%)	541 (7.9%)	
PS	11 (0.2%)	0	11 (0.2%)	0.495
PI grades				0.929
None	6881 (96.1%)	277 (95.5%)	6604 (96.1%)	
Mild	253 (3.5%)	12 (4.1%)	241 (3.5%)	
Moderate	26 (0.4%)	1 (0.3%)	25 (0.4%)	
Severe	3 (0.0%)	0	3 (0.0%)	
Intraoperative factors				
CPB time (min)	129.2 ± 62.3	196.4 ± 137.4	126.4 ± 55.4	<0.001
ACC time (min)	87.3 ± 42.0	113.1 ± 66.1	86.4 ± 40.4	<0.001
Combined CABG	1765 (24.6%)	114 (39.3%)	1651 (24.0%)	<0.001
Aortic valve surgery	3757 (52.5%)	126 (43.4%)	3631 (52.8%)	0.007
Mitral valve surgery	4354 (60.8%)	203 (70.0%)	4151 (60.4%)	0.002
Tricuspid valve surgery	2623 (36.6%)	115 (39.7%)	2508 (36.5%)	0.461
Pulmonary valve surgery	88 (0.0%)	4 (0.0%)	84 (0.0%)	0.838
Others	2175 (30.4%)	109 (37.6%)	2066 (30.1%)	0.006
RBC transfusion (u)	2 (1.5,4)	4 (2,8)	2 (1,4)	<0.001
FFP transfusion (u)	2 (0,3)	2.5 (1.5,5)	2 (0,3)	<0.001
Postoperative factors				
RBC transfusion (u)	2 (1,5)	8 (3,16)	2 (1,4.5)	<0.001
FFP transfusion (u)	2 (1,4)	4.5 (2,12)	2 (1,4)	<0.001
Mechanical ventilation time (h)	20 (15,33)	76 (22,203)	20 (15,29)	<0.001
Reintubation	152 (2.5%)	60 (20.7%)	92 (1.3%)	<0.001
Initial ICU stays (h)	66 (42,96)	120 (45,253)	66 (42,96)	<0.001
Readmission to the ICU	176 (2.5%)	45 (15.5%)	131 (1.9%)	<0.001
Readmission ICU stays (h)	83 (31,179)	75 (18,293)	84 (38,141)	0.856
Volume of drainage (ml)	560 (0.1020)	1200 (395,2218)	550 (0.990)	<0.001
Reoperation	280 (3.9%)	60 (20.7%)	220 (3.2%)	<0.001
Cardiac tamponade	40 (0.6%)	10 (3.4%)	30 (0.4%)	<0.001
Postoperative MI	50 (0.7%)	5 (1.7%)	45 (0.7%)	0.032
New-onset cerebrovascular accident	27 (0.4%)	10 (3.4%)	17 (0.2%)	<0.001
Pulmonary embolism	2 (0.0%)	2 (0.7%)	0 (0.0%)	<0.001
Acute kidney injury	156 (2.2%)	88 (30.3%)	68 (1.0%)	<0.001
Dialysis	100 (1.4%)	69 (23.8%)	31 (0.5%)	<0.001
New-onset atrial fibrillation	158 (2.2%)	16 (5.5%)	142 (2.1%)	<0.001
MODS	111 (1.5%)	106 (36.6%)	5 (0.1%)	<0.001

Continuous variables are presented as medians with quartiles or means with standard deviation. Frequencies with proportions are displayed for categorical variables. The number in parenthesis represents the number of patients with available data. * *p* < 0.05. BMI, body mass index; CKD, chronic kidney disease; COPD, chronic obstructive pulmonary disease; PVD, peripheral vascular disease; HF, heart failure; CCS, Canadian Cardiovascular Society; NYHA, New York Heart Association; MI, myocardial infarction; PCI, percutaneous transluminal coronary intervention; CABG, coronary artery bypass grafting; SCr, serum creatinine; CCr, creatinine clearance rate; LDL, low density lipoprotein; FBG, fasted blood glucose; LVEF, left ventricular ejection fraction; LVEDD, left ventricular end-diastolic diameter; LAD, left atrial dimension; AS, aortic stenosis; AI, aortic insufficiency; MS, mitral stenosis; MI, mitral insufficiency; TS, tricuspid stenosis; TI, tricuspid insufficiency; PS, pulmonary stenosis; PI, pulmonary insufficiency; CPB, cardiopulmonary bypass; ACC, aortic cross-clamp; ICU, intensive care unit; MODS, multiple organ dysfunction syndrome.

**Table 3:** Multivariable LASSO-logistic regression analysis of perioperative parameters.

Risk Factors	Coefficient	Odds Ratio	95% CI		*p* Value *
			LCI	UCI	
Age	0.036	1.037	1.011	1.063	0.005
Prior cardiac surgery	0.928	2.529	1.572	4.070	0.000
LVEF	−0.026	0.974	0.957	0.991	0.003
CCr	−0.021	0.979	0.970	0.989	0.000
CPB time	0.01	1.010	1.009	1.012	0.000
Combined CABG	0.389	1.475	1.038	2.097	0.03
NYHA class	0.328	1.389	1.090	1.769	0.000

The LASSO-logistic regression analysis was established based on Forward LR. *, *p* < 0.05. LVEF, left ventricular ejection fraction; CCr, creatinine clearance rate; CPB, cardiopulmonary bypass; CABG, coronary artery bypass grafting; NYHA, New York Heart Association; CI, confidence interval; LCI, lower confidence interval; UCI, upper confidence interval.

## Data Availability

The data presented in this study are available upon request from the corresponding author. The data are not publicly available due to the No.717 Chinese Decree of the State Council, suggesting that the raw data of large samples cannot be shared globally.

## References

[1] Coffey S., Roberts-Thomson R., Brown A., Carapetis J., Chen M., Enriquez-Sarano M., Zühlke L., Prendergast B.D. (2021). Global epidemiology of valvular heart disease. Nat. Rev. Cardiol..

[2] Yadgir S., Johnson C.O., Aboyans V., Adebayo O.M., Adedoyin R.A., Afarideh M., Alahdab F., Alashi A., Alipour V., Arabloo J. (2020). Global, Regional, and National Burden of Calcific Aortic Valve and Degenerative Mitral Valve Diseases, 1990–2017. Circulation.

[3] Ambler G., Omar R.Z., Royston P., Kinsman R., Keogh B.E., Taylor K.M. (2005). Generic, Simple Risk Stratification Model for Heart Valve Surgery. Circulation.

[4] O’Brien S.M., Shahian D.M., Filardo G., Ferraris V.A., Haan C.K., Rich J.B., Normand S.L., DeLong E.R., Shewan C.M., Dokholyan R.S. (2009). The Society of Thoracic Surgeons 2008 cardiac surgery risk models: Part 2—Isolated valve surgery. Ann. Thorac. Surg..

[5] Yang Y., Wang Z., Chen Z., Wang X., Zhang L., Li S., Zheng C., Kang Y., Jiang L., Zhu Z. (2021). Current status and etiology of valvular heart disease in China: A population-based survey. BMC Cardiovasc. Disord..

[6] The Writing Committee of the Report on Cardiovascular Health and Diseases in China (2022). Report on Cardiovascular Health and Diseases in China 2021: An Updated Summary. Biomed. Environ. Sci..

[7] Jamieson W.R.E., Edwards F.H., Schwartz M., Bero J.W., Clark R.E., Grover F.L. (1999). Risk stratification for cardiac valve replacement. National Cardiac Surgery Database. Ann. Thorac. Surg..

[8] Nashef S.A.M., Roques F., Michel P., Gauducheau E., Lemeshow S., Salamon R., the Euro Ssg (1999). European system for cardiac operative risk evaluation (EuroSCORE). Eur. J. Cardio-Thorac. Surg..

[9] Nashef S.A.M., Roques F., Sharples L.D., Nilsson J., Smith C., Goldstone A.R., Lockowandt U. (2012). EuroSCORE II†. Eur. J. Cardio-Thorac. Surg..

[10] Zheng Z., Zhang L., Li X., Hu S., on behalf of the Chinese CRS (2013). SinoSCORE: A logistically derived additive prediction model for post-coronary artery bypass grafting in-hospital mortality in a Chinese population. Front. Med..

[11] Xu H., Liu Q., Cao K., Ye Y., Zhang B., Li Z., Hao J., Qi X., Zhao Q., Liu S. (2022). Distribution, Characteristics, and Management of Older Patients With Valvular Heart Disease in China: China-DVD Study. JACC Asia.

[12] Rao C., Zhang H., Gao H., Zhao Y., Yuan X., Hua K., Hu S., Zheng Z. (2016). The Chinese Cardiac Surgery Registry: Design and Data Audit. Ann. Thorac. Surg..

[13] Kodali S.K., Velagapudi P., Hahn R.T., Abbott D., Leon M.B. (2018). Valvular Heart Disease in Patients ≥ 80 Years of Age. J. Am. Coll. Cardiol..

[14] Iung B., Vahanian A. (2011). Epidemiology of valvular heart disease in the adult. Nat. Rev. Cardiol..

[15] Yoshida K., Yoshikawa J., Akasaka T., Shakudo M., Jyo Y., Takao S., Shiratori K., Koizumi K., Okumachi F., Kato H. (1988). Problems in the management of elderly patients with valvular heart disease. Jpn. Circ. J..

[16] Hu Z., Chen S., Du J., Gu D., Wang Y., Hu S., Zheng Z. (2020). An In-hospital Mortality Risk Model for Patients Undergoing Coronary Artery Bypass Grafting in China. Ann. Thorac. Surg..

[17] Zhuge R.Q., Hou X.P., Qi X.L., Wu Y.J., Zhang M.Z. (2018). Clinical features and treatment options for mitral regurgitation in elderly inpatients. J. Geriatr. Cardiol..

[18] Mack M.J. (2011). Risk Scores for Predicting Outcomes in Valvular Heart Disease: How Useful?. Curr. Cardiol. Rep..

[19] Nowicki E.R., Birkmeyer N.J., Weintraub R.W., Leavitt B.J., Sanders J.H., Dacey L.J., Clough R.A., Quinn R.D., Charlesworth D.C., Sisto D.A. (2004). Multivariable prediction of in-hospital mortality associated with aortic and mitral valve surgery in Northern New England. Ann. Thorac. Surg..

[20] Lin H., Gong J., Wu Y., Zheng Z., Hou J. (2022). A Comparative Study on Surgical Treatment of Valvular Heart Disease between High-Volume Cardiac Centers in China and STS Data. J. Cardiovasc. Dev. Dis..

[21] Salis S., Mazzanti V.V., Merli G., Salvi L., Tedesco C.C., Veglia F., Sisillo E. (2008). Cardiopulmonary bypass duration is an independent predictor of morbidity and mortality after cardiac surgery. J. Cardiothorac. Vasc. Anesth..

[22] Ascione R., Lloyd C.T., Underwood M.J., Lotto A.A., Pitsis A.A., Angelini G.D. (2000). Inflammatory response after coronary revascularization with or without cardiopulmonary bypass. Ann. Thorac. Surg..

[23] Sirvinskas E., Andrejaitiene J., Raliene L., Nasvytis L., Karbonskiene A., Pilvinis V., Sakalauskas J. (2008). Cardiopulmonary bypass management and acute renal failure: Risk factors and prognosis. Perfusion.

[24] Murphy G.J., Angelini G.D. (2004). Side effects of cardiopulmonary bypass: What is the reality?. J. Card. Surg..

[25] Chen J.H., Asch S.M. (2017). Machine Learning and Prediction in Medicine—Beyond the Peak of Inflated Expectations. N. Engl. J. Med..

[26] Deo R.C. (2015). Machine Learning in Medicine. Circulation.

[27] Rajkomar A., Dean J., Kohane I. (2019). Machine Learning in Medicine. N. Engl. J. Med..

[28] Nilsson J., Ohlsson M., Thulin L., Höglund P., Nashef S.A.M., Brandt J. (2006). Risk factor identification and mortality prediction in cardiac surgery using artificial neural networks. J. Thorac. Cardiovasc. Surg..

[29] LaFaro R.J., Pothula S., Kubal K.P., Inchiosa M.E., Pothula V.M., Yuan S.C., Maerz D.A., Montes L., Oleszkiewicz S.M., Yusupov A. (2015). Neural Network Prediction of ICU Length of Stay Following Cardiac Surgery Based on Pre-Incision Variables. PLoS ONE.

[30] Thottakkara P., Ozrazgat-Baslanti T., Hupf B.B., Rashidi P., Pardalos P., Momcilovic P., Bihorac A. (2016). Application of Machine Learning Techniques to High-Dimensional Clinical Data to Forecast Postoperative Complications. PLoS ONE.

[31] Shameer K., Johnson K.W., Glicksberg B.S., Dudley J.T., Sengupta P.P. (2018). Machine learning in cardiovascular medicine: Are we there yet?. Heart.

[32] Allyn J., Allou N., Augustin P., Philip I., Martinet O., Belghiti M., Provenchere S., Montravers P., Ferdynus C. (2017). A Comparison of a Machine Learning Model with EuroSCORE II in Predicting Mortality after Elective Cardiac Surgery: A Decision Curve Analysis. PLoS ONE.

